# Exercise as a Peripheral Circadian Clock Resynchronizer in Vascular and Skeletal Muscle Aging

**DOI:** 10.3390/ijerph182412949

**Published:** 2021-12-08

**Authors:** Bruna Spolador de Alencar Silva, Juliana Souza Uzeloto, Fábio Santos Lira, Telmo Pereira, Manuel J. Coelho-E-Silva, Armando Caseiro

**Affiliations:** 1 Exercise and Immunometabolism Research Group, Universidade Estadual Paulista (UNESP), Presidente Prudente, São Paulo 19060-560, Brazil; brunaspolador@gmail.com (B.S.d.A.S.); juliana_uzeloto@hotmail.com (J.S.U.); 2 Clinical Physiology, Coimbra Health School, Polytechnic Institute of Coimbra, Rua 5 de Outubro—SM Bispo, Apartado 7006, 3046-854 Coimbra, Portugal; telmo@estescoimbra.pt; 3 Laboratory for Applied Health Research (LabinSaúde), Rua 5 de Outubro—SM Bispo, Apartado 7006, 3046-854 Coimbra, Portugal; 4 Faculty of Sport Science and Physical Education, University of Coimbra, 3004-531 Coimbra, Portugal; mjcesilva@hotmail.com; 5 Unidade I&D Química-Física Molecular, Faculdade de Ciências e Tecnologia, Universidade de Coimbra, 3004-535 Coimbra, Portugal; 6 Biomedical Laboratory Sciences, Coimbra Health School, Polytechnic Institute of Coimbra, Rua 5 de Outubro—SM Bispo, Apartado 7006, 3046-854 Coimbra, Portugal

**Keywords:** sarcopenia, inflammation, circadian rhythms, clock genes, vascular disfunction, skeletal muscle disfunction

## Abstract

Aging is characterized by several progressive physiological changes, including changes in the circadian rhythm. Circadian rhythms influence behavior, physiology, and metabolic processes in order to maintain homeostasis; they also influence the function of endothelial cells, smooth muscle cells, and immune cells in the vessel wall. A clock misalignment could favor vascular damage and indirectly also affect skeletal muscle function. In this review, we focus on the dysregulation of circadian rhythm due to aging and its relationship with skeletal muscle changes and vascular health as possible risk factors for the development of sarcopenia, as well as the role of physical exercise as a potential modulator of these processes.

## 1. Introduction

Aging is characterized by several progressive physiological alterations, such as hormonal imbalance, impaired proteostasis, mitochondrial dysfunction, and cellular senescence, which lead to functionality loss and increased risk of death [1]. The circadian rhythm also undergoes significant age-related disturbances, which can contribute to the development of several morbidities [2].

Circadian rhythms involve biological rhythms that work by the interaction of several exogenous and endogenous factors, which together influence behavior, physiology, and metabolic processes in order to maintain homeostasis. The suprachiasmatic nucleus (SCN), together with other autonomous clocks, present in virtually all cells of the body, is responsible for controlling the central and peripheral circadian rhythm. However, the so-called synchronizers (also known as “zeitgebers”), such as external factors, can influence the functioning of these rhythms. The main synchronizer for SCN is the light–dark cycle that, through the retinohypothalamic tract, provides information to the SCN, which, by means of neurohumoral signaling and core body temperature oscillating, leads to the synchronization of peripheral clocks [3]. However, there are also non-photic synchronizers such as food, physical activity, and stress [4].

Two main elements control the activity of the molecular clock—circadian locomotor output cycles kaput (CLOCK) and brain and muscle ARNT-like protein 1 (BMAL1), which activate the transcription of the period 1,2 and 3 (Per) proteins and cryptochrome 1 and 2 (Cry) that, when migrating to the cell nucleus, inhibit the initial dimerization of CLOCK-BMAL1, closing a cycle of approximately 24 h. The circadian oscillation of BMAL1 is also up- and downregulated by retinoid-related orphan receptors RORs (α, β, and γ) and REV-ERBs (α and β), respectively [5]. This daily self-sustaining circuit of gene expression controls several physiological processes, essential for the organism [3].

Some physiological changes related to the aging process favor central and peripheral rhythms impairment [6] such as reduction in the total secretion of melatonin [2,7] and metabolism-related rhythms (glucose control, lipid metabolism, and xenobiotic detoxification), controlled by clocks in the liver and pancreas, which can contribute to the development of metabolic diseases [8].

In muscle tissue, endogenous clock alteration can influence myogenic capacity, making it difficult to transcribe mTOR and impairing the development and growth of skeletal muscles [9]. Evidence shows that the loss of CLOCK-BMAL1 molecular rhythm function is associated with metabolic inefficiency with protein catabolism and the replacement of glucose metabolism by lipids [10,11]. Still, there may be changes in the types of muscle fiber, reduction in mitochondria, and damage to mitochondrial breathing, and in the structure and function of sarcomeres, causing severe muscle damage [8,12]. Therefore, clock gene misalignment alters muscle composition and function, which has a strong association with health status, with low strength and muscle mass being independent factors for the development of sarcopenia, which is strongly associated with breaking the circadian rhythm regardless of age; in the elderly, this factor is enhanced, which leads to functional incapacities, falls, osteoporosis, dyslipidemia, increased cardiovascular risk, metabolic syndrome, immunosuppression, and increased risk of mortality [13].

An inherent factor in aging that can also affect clock genes and muscle functions is chronic low-grade inflammation. Among other factors, it can be developed as a result of the immunosenescence process. It is known that the senescence of immune cells can reflect changes in skeletal muscle function, such as muscle regeneration and repair [14,15]. In addition, senescent immune cells accumulated during aging generally express senescence-associated inflammatory factors (SASP) with overexpression of IL1β, IL-6, IL-8, TNF-α, IFN-γ, etc. [16]. These factors associated with resident muscle cells can also modulate the expression of other surface molecules in muscle cells and promote an inflammatory environment [14], which, in turn, can negatively influence the local clock, making it a vicious cycle.

It is suggested that the decrease in vascular health also causes clock activity in the skeletal muscle. There is a decrease in vascular health with aging, such as increased stiffness of the large elastic arteries and endothelial dysfunction [17]. The dysfunction of peripheral arteries decreases blood flow to the body extremities, which can lead to decreased calf muscle area and strength [18]. Circadian rhythms also influence the function of endothelial cells, smooth muscle cells, and immune cells in the vessel wall, so a clock misalignment could favor vascular damage (e.g., impair vessel contractility and endothelial integrity [19]) and indirectly also affect skeletal muscle function.

**Figure 1:**
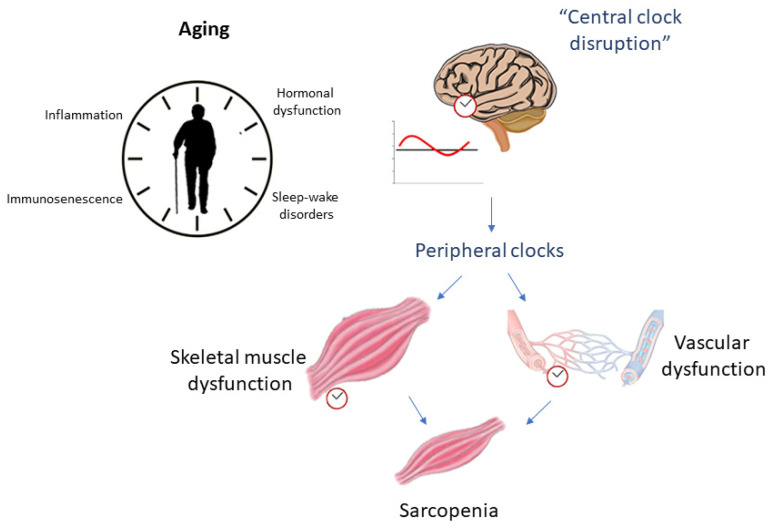
Aging relationship with circadian rhythm disruption as possible influencers of sarcopenia.

**Figure 2:**
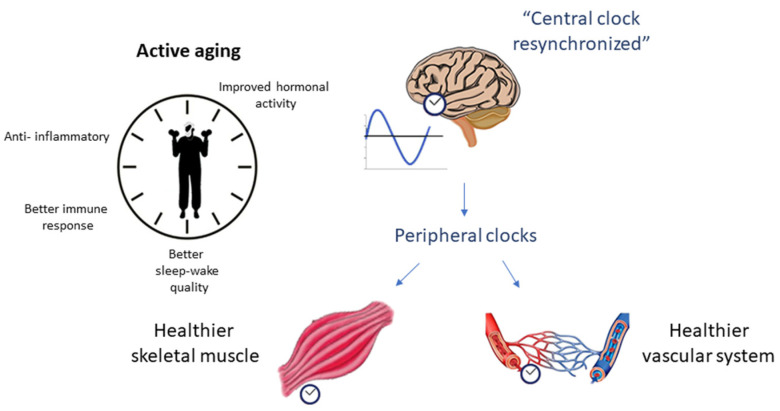
The role of physical exercise as a potential positive modulator of circadian rhythm and sarcopenia.

In this sense, several studies attempt to elucidate the role of circadian rhythm in the inflammatory process (and vice versa) related to aging. In this review, we focus on the dysregulation of circadian rhythm due to aging (mainly from an inflammatory perspective) and its relationship with skeletal muscle changes and vascular health as possible risk factors for the development of sarcopenia (Figure 1), as well as the role of physical exercise as a potential modulator of these processes (Figure 2).

## 2. Sarcopenia: Concept and Relationship with Inflammation

The reduction in muscle strength and mass are important morphological changes that occur as a result of aging, and when associated, characterize a muscle disease called sarcopenia [20]. Sarcopenia is associated with a greater likelihood of adverse outcomes, such as falls, fractures, physical disability, development of chronic diseases, and mortality [20]. Primarily, its development is related to aging, but other causal factors contributing to the disease include a sedentary lifestyle, inadequate nutrition, and inflammation [21,22,23].

Chronic low-grade inflammation is characterized by an increase in circulating concentrations of tumor necrosis factor α (TNF-α), interleukin 6 (IL-6), IL-1 β, etc., and is present in numerous chronic diseases [24,25,26], including sarcopenia [27]. Among the causal factors of inflammation associated with aging (inflammaging) are the accumulation of adipose tissue, mainly visceral adipose tissue (VAT), common in the elderly due to hormonal changes, unbalanced diet, and reduced physical activity [28]. VAT produces and releases pro-inflammatory cytokines and chemokines, influencing, in addition to body weight, the onset of and increase in inflammation [29]. Another causal factor of inflammation associated with age may be the decline in the functionality and efficiency of immune cells during the aging process, called immunosenescence [30]. The constant ineffective signaling of senescent cells favors a continuous stimulus for the inflammatory response in the elderly [31].

High plasma concentrations of pro-inflammatory cytokines, such as IL-6 and TNF-α, among other disorders, are associated with lower strength and MM in the elderly [32], as they provoke the stimulation of protein catabolism and suppression of muscle synthesis [27,33,34]. Positive regulation of TNF-α activates the expression of proteolytic enzymes (e.g., muscle ring finger1 (MuRF1) and atrogin-1) that, via ubiquitin-proteasome (UbP) or mitogen-activated p38 protein kinase (p38MAPK), increase protein degradation [35,36,37]. IL-6 already acts in a pleiotropic manner, and when expressed by immune cells (such as CD4/CD8-TEMRA cells), it has a pro-inflammatory function that can impair muscle anabolism and energy homeostasis, and directly mediate muscle catabolism through STAT3-IL-6 signaling [14,38]. However, when expressed through muscle contraction, it has an anti-inflammatory function, by promoting the increase in an important anti-inflammatory cytokine, IL-10 [39]. IL-10 can also be expressed in response to the presence of overregulation of cytokines pro-inflammatory, blocking (via inhibition of the transcription factor NF-kB) the expression of pro-inflammatory cytokines, such as TNF-α, IL-6, etc. [40].

### 2.1. Crosstalk between Skeletal Muscle and Immune Cells

Skeletal muscle remodeling is dependent on the interaction between skeletal muscle and immune cells; thus, the immunosenescence process alone can also negatively influence skeletal muscle morphology and regenerative capacity [14]. Although the skeletal muscle has “resident” immune cells, after an acute effect, such as injury, the peripheral blood circulation immune cells are recruited into the muscle by chemotactic signals to assist tissue repair [41]. The signaling of CXCL1 or CXCL5 by muscle cells are potential candidates to act as early activators of the inflammatory response to acute muscle injury [42]. Both chemokines are chemoattractive to neutrophils, which rise significantly in the muscle shortly after injury and help to boost the inflammatory response.

Neutrophils are the first immune cells that infiltrate muscle lesions, and their presences are required for an optimal muscle regeneration process [42]. Following neutrophil infiltration, there is a monocyte migration in response to chemokines secreted. At this moment, the expressed cytokines characterize a Th1 response (e.g., interferon-γ (IFN-γ) and TNF-α) that lead to the activation of macrophages to an M1 (pro-inflammatory) phenotype capable of continuing the inflammatory response. Macrophages, in addition to attracting satellite cells to the injury site and stimulating their proliferation, also produce cytokines (e.g., TNF-α, IL-6) that also stimulate satellite cell proliferation [43]. After the M1 macrophages perform their function and reach their peak, they are replaced by M2 macrophages (anti-inflammatory) that act to decrease inflammation and promote tissue repair. These are activated by Th2 cytokines, interleukin-4 (IL-4), IL-13 (wound healing and tissue repair), IL-10 (inhibition of the M1 phenotype), or by other molecules that enable the release of anti-inflammatory cytokines [44]. T lymphocytes also influence other immune cells or muscle satellite cells and play a pleiotropic role in the process of muscle regeneration and repair [14,15]. T cells affect the proliferation and migration of satellite muscle cells [45]. In damaged muscles, CD8+ T cells stimulate the recruitment of macrophages, thus promoting the proliferation of myoblasts [46], and regulatory T cells can release growth factors and promote muscle growth in response to specific cytokines [47].

In this sense, temporal immune response (acute inflammation) is necessary for muscle stem cell activation and proliferation during regeneration; however, a continued immune response, as explained above (Section 2), triggers protein catabolism and impairments anabolic processes of skeletal muscle in the long-term leading to sarcopenia [48]. With aging, there may be a possibility of prolonged accumulation of neutrophils in skeletal muscle during muscle recovery, an abundance of inflammatory monocytes, and impaired macrophage polarization, which would affect their function in muscle repair [49]. The impaired signaling of some aging-related cytokines can also significantly impact the immune system and consequently the skeletal muscle. The impairment in IL-15 signaling impairs the proliferation and survival of naïve T cells on CD8 T cells, as well as the migration and phagocytosis of neutrophils [14]. Impairment in IL-7 signaling affects the development and maintenance of T- and B lymphocytes, failing to support the thymic function that has already decreased with age [50]. In addition, senescent T cells accumulated during aging usually overexpress pro-inflammatory cytokines [16], which, in addition to enhanced skeletal muscle wasting, can modulate the expression of other surface molecules in muscle cells and favor a possible establishment of a local inflammatory environment [14]. Furthermore, the impairment of immune cell function as a result of aging itself affects skeletal muscle repair function, which can also contribute to the development of sarcopenia.

### 2.2. Inflammation and Circadian Misalignment: A Two-Way Road in Contributing to Sarcopenia

Chronic low-grade inflammation could also influence the dysregulation of circadian rhythm controlling genes. Studies indicate that cytokines affected the expression of core clock genes expressed by the peripheral clocks, despite the intrinsic mechanisms are still unknown. Cavadini et al. suggested that pro-inflammatory cytokines (TNF-alpha and IL-1beta) impair the function of clock genes in fibroblasts of mice [51]. In synovial cells, TNF-alpha promotes an increase in the expression of BMAL1 and Rorα, while decreasing Rev-erbα [52].

Conversely, the deregulation of clock genes also affects inflammation. Deletion of clock genes in macrophages induces upregulation of pro-inflammatory cytokines production, which can be accompanied by a rise in oxidative stress [53]. In BMAL1-deficient mice, there is a pro-inflammatory increase due to upregulation of NF-κB-mediated by CLOCK, which may result in chronic inflammation [54]. In the same sense, the absence of Cry proteins upregulates the expression of pro-inflammatory cytokines, through NF-κB activation through phosphorylation of p65 [55]. Studies show that BMAL1 KO mice have a short lifespan, have advanced aging phenotypes, and favor the emergence of chronic diseases [56]. In this sense, the power of clock genes on the inflammatory profile (and vice versa) can be a key point for the development of treatment strategies for the adverse effects of aging.

The age-related circadian rhythm disruption relationship with inflammation could indirectly promote negative influence in skeletal muscle tissue, in addition to direct disorders in the skeletal muscle intrinsic molecular clock itself. Several studies have found specific changes in clock genes with different muscle disorders, including changes in structural and metabolic processes (decreased glucose uptake and insulin sensitivity, impaired oxidative capacity, mitochondrial decrease, atrophy, and impaired regeneration (reviewed in [9])) As examples, deficiency in BMAL1 clock gene promotes severe sarcopenia with age [57]. BMAL1 and Sirtuin1 (SIRT1) are downregulated in skeletal muscle of mice maintained in constant abstaining from light [58]. Furthermore, regardless of age, circadian rhythm disruption associated with shift work may contribute to an increased risk of sarcopenia [59]. In this sense, muscle clock genes regulate the expression of several genes that act on the local metabolism, and a change in these clocks could cause losses in insulin sensitivity and loss of muscle mass, contributing to the development of sarcopenia [8,58].

Despite the current understanding of the role of the molecular clock in preventing age-related sarcopenia, investigations into the potential modulating effect of physical exercise on physiological mechanisms, including the maintenance of skeletal muscle growth and function, are emerging.

### 2.3. Vascular Disfunction and Sarcopenia

One of the most noteworthy expressions of biological aging is vascular aging, and arterial stiffness (AS) is currently an established independent risk factor for cardiovascular disease (CVD), above and beyond conventional risk factors [60]. Several studies have documented a close relation between sarcopenia and AS [61], and an interplay between AS, sarcopenia, and cognitive impairment in the elderly has also been suggested [62]. The link between sarcopenia and vascular dysfunction may even be aggravated by the clustering of cardiovascular risk factors in old adults, which impair blood circulation and muscle supply, constituting an added factor for impaired muscle function and overall functionality in the elderly population [63].

The link between vascular dysfunction and sarcopenia can be examined at the micro and macrovascular levels, with intersecting contributions that add to significant muscle effects, and may share common denominators, e.g., endothelial dysfunction and vascular calcification [64].

Vascular calcification follows aging as a result of the long-lasting stress that is imposed hemodynamically onto the arterial wall. Its distribution may include the inner layers of the arterial wall or even spread onto the outer layers, promoting changes in the arterial wall dynamics, which, in turn, produces downstream hemodynamic changes germane to inadequate muscle perfusion [65,66]. Several biological mechanisms have been implied in age-dependent vascular calcification. Oxidative stress is an all-mark in aging and has a well-established role in vascular dysfunction. Reactive oxygen species yields endothelial cell apoptosis, interferes with nitric oxide (NO), and unbalances monocyte dynamics, leading to endothelial dysfunction and impaired endothelial-dependent vasodilation [67,68]. Deposition of calcium in the arterial wall as a consequence of vascular smooth muscle cells senescence and osteochondrogenesis is also related to oxidative-stress-induced hyperphosphatemia through the promotion of p65 translocation [69,70]. Oxidative stress is also a cornerstone for a proinflammatory status in the circulatory system, activating the NF-κB, which, in turn, increases the release of proinflammatory cytokines, decisively contributing to atherosclerosis and calcification [71,72]. Circulating cytokines are known to promote calcification through TNF-α, which interferes with the MGP and induces mineral deposition in the atheroma plaque encompassed in the atherosclerotic continuum [73] vis-a-vis the stimulation of smooth muscle cell differentiation onto osteoblast-like configurations [74]. Increasing blood pressure further enhances these effects, combining a mechanical substrate in the arterial wall with neurohumoral paths that further damages and stiffens the arterial wall [60,65]. Aside from the shear-stress aggression promoted by high blood pressure, insulin resistance is also a crucial contributor to vascular dysfunction and calcification by reducing NO bioavailability [75].

An inverse relation between micro- and macrovascular dysfunction and skeletal muscle mass and function has been recently depicted in a systematic review including 33 clinical studies [63]. It is believed that this association between vascular dysfunction and muscle loss (mass and function) may be associated with impaired muscle perfusion through a reduction in peripheral blood flow, relying both on anatomic changes and hemodynamic features of the aging circulatory physiology. The blood flow restrictions to the muscles will limit the supply of important nutrients and hormones to the myocytes, from which muscle function and structure will be affected and progress in close connection with sarcopenia. In fact, it is suggested that this reduction in the nutritive supply may contribute to muscle dysfunction and loss through changes in anabolic resistance of the muscle (e.g., [76]).

The identification and implementation of strategies to prevent and treat these features are of the utmost importance. These may include aspects such as proper management of behavioral and environmental risk factors, such as nutrition, exercise, smoking habits, stress management, social support, pollution, and body composition. Physical exercise should play a major role, as it is the main component for the prevention and treatment of sarcopenia [77], and further evidence exists that additionally supports the positive modulation of physical exercise for vascular health [78,79] in old adults. Therefore, physical activity should contribute to a positive modulation of the aging trajectories, particularly if administered in a personalized approach, tailored to the individual needs and expectations.

## 3. Impact of Exercise on Inflammatory Profile and Association with Clock Genes

Exercise, specifically aerobic and combined with resistance training, have been recognized as a potential intervention to modulate inflammatory profile in humans [80,81,82], with a particular emphasis in older adults, in reversing or attenuate immunosenescence in aging [83,84,85], as well as reducing the development of chronic diseases [86].

During exercise, skeletal muscle functions as an endocrine organ, secreting several myokines such as IL-6, IL-7, and IL-15. In addition to the pleotropic effects mentioned above, IL-6 can stimulate cortisol released by the adrenal glands, acting as a second anti-inflammatory signal and improving glucose uptake through the stimulation of AMPK signaling [87]. IL-6 is conventionally used as marker of inflammation and several studies evaluating the impact of exercise in their inflammatory profile report the significant reduction in IL-6 levels after different periods of intervention. A study on females with metabolic syndrome found that a 12 week-long aerobic exercise intervention promotes a decrease in IL-6 [88]. The role of IL-6 in the framework of exercise as an anti-inflammatory therapy for cancer cachexia is also very significant (reviewed in [86]).

A study conducted by Chen et al. [89] performed an evaluation of the differentially expressed genes (DEGs) in a group of 24 sedentary middle-aged men with different basal levels of IL-6 that undertook a 24 week-long physical activity program. The analysis of DEGs, followed by functional enrichment analysis and protein–protein interactions, showed that C-C motif chemokine receptor 7 (CCR7) and hemoglobin subunit delta (HBD) genes were induced by myocyte enhancer factor 2A (MEF2A), arising as key regulatory factors modulated by exercise [89]. The authors conclude that inflammation-related genes such as CCR7, HBD, and interferon-gamma (IFN-γ) might serve vital roles in reducing inflammation by exercise and might prevent the risk of chronic diseases in sedentary individuals [89].

IFN-γ is a cytokine with a relevant role in several aspects of both adaptive and innate immunity. Firstly, it is most recognized for its pro-inflammatory properties but has been also recognized for its pleiotropic functions such as induction and maintenance of regulatory T cells, induction of tolerogenic dendritic cell characteristics, and immunosuppressive tumor environment. These properties place IFN-γ among the major endogenous immune regulators, contributing to both immunity and tolerance in several stages of the immune response [90,91]. Svajger et al. (2021) demonstrated that IFN-γ can exert important tolerogenic effects on dendritic cells, in in vitro assays, through a strong upregulation of programmed death-ligand 1 (PD-L1), an inhibitory molecule [90].

Shaw et al. (2020) observed that exercise in acute hyperketonemia appears to amplify the initiation of the pro-inflammatory T-cell-related IFN-γ response, with an increased IFN-γ mRNA expression during and following prolonged, strenuous exercise [92]. Hasanli et al. (2020) evaluated the impact of physical or psychological stress on the IFN-γ levels in male Sprague Dawley rats submitted to exercise activity, psychological stress, or the combination of exercise and psychological stress. The study showed that the different interventions did not modulate significantly the levels of IFN-γ either immediately after exercise or after 72 h, despite fluctuations in cytokine values, with a tendency to decrease immediately after exercise and increase 72 h later [93]. Vijayaraghava (2017) conducted an experimental study with the application of different grades of exercise and evaluated the impact on IFN-γ plasma levels, in individuals of different ages and body mass index (BMI) values. The levels of plasma IFN-γ were significantly modulated by moderate exercise, with a significant increase in plasma levels after a bout of moderated exercise, and the highest values were found after 1 month of moderate exercise. On the contrary, after a bout of strenuous exercise, plasma levels reduced in comparison with baseline values. The study results also showed that regular physical activity confers protection against excessive inflammation in spite of higher age or BMI, with respect to IFN-γ levels [94]. Conversely, Farinha et al. evaluated the impact of 12 weeks of aerobic exercise and observed a decrease in IL-1β, TNF-α, IL-6, and IFN-γ in women with metabolic syndrome [88].

Therefore, exercise has an emerging potential to improve immune system performance and reduce the risk of low-grade inflammation from childhood to old age [88,95,96,97,98,99]. A systematic review conducted by Bautmans et al. (2021) revealed significant anti-inflammatory effects of exercise in the elderly—namely, in reducing circulating levels of CRP, IL-6, and TNF-alpha and also that the performed exercise interventions seem suitable to apply and safe for older patients, without inducing an exacerbation of inflammation following exercise [100].

Desynchronization of circadian clocks induced by modern lifestyle could predispose to inflammation and metabolic impairment and increase the risk of chronic diseases [101,102]. A relevant aspect is the connection of exercise with circadian rhythm physiology. Several studies point that exercise can modulate circadian rhythm, acting as a circadian time cue and changing the phase of a molecular clock in peripheral tissues. In addition to studies on animal models, studies on humans have revealed that endurance and resistance exercises stimulate the expression of clock genes [103,104,105]. Several studies explain the effect of exercise on core molecular clock genes, through the influence in exercise-responsive genes—AMPK, HIF-1α, and PGC1α. Increased activity of AMPK changes Per and Cry stability, modulating clock genes expression [103,105,106].

A recent study by Souza Teixeira et al. shows an improvement in the anti-inflammatory profile, with a lifelong physical exercise related to clock genes expression in effector-memory CD4+ T cells in master athletes. Master athletes presented different peripheral and cellular inflammatory responses after acute exercise, compared with untrained individuals, with higher levels of IL-8, IL-10, IL-12p70, and IL-17A and augmented expression of Cry1, REV-ERBα, and TBX21 [107].

Clock genes are involved in inflammatory response through the activation of NF-κB transcription and activation of pro-inflammatory cytokines [107,108]. CLOCK can upregulate NF-κB-mediated transcription in the absence of BMAL1; therefore, BMAL1 may have an anti-inflammatory role [54]. Tylutka et al. concluded that physical activity sustained throughout life could lead to rejuvenation of the immune system by increasing the percentage of naïve T lymphocytes or by decreasing the inverse CD4/CD8 ratio [108]. Taking into account the available data, growing evidence supports the vision that exercise may counteract immunosenescence and improve the immune system. Exercise-induced changes in immunosenescence-related markers of immune cells were reviewed by Mathot et al., supporting the effect of long-term exercise on senescent T-lymphocytes and the increase in dendritic cells after exercise in older adults. The data also suggest a significant influence of the type and intensity of exercise on immunosenescence-related markers, mainly in older adults, highlighting the greater impact of aerobic exercise and resistance exercise protocols with lower loads and a greater number of repetitions (2 sets of 30 consecutive repetitions at 40% of 1RM) [85,107].

## 4. Impact of Exercise on Circadian Skeletal Muscle Rhythm

The skeletal muscle system has its own clock gene expression and can be stimulated by physical activity. Acute aerobic and resistance exercise increases the expression of skeletal muscle clock genes in humans [109]. An acute session of aerobic exercise (70 min at 70% VO2max) increased the expression of the BMAL1 gene by 1.6 times 4 h after exercise, and to 3.5 times 8 h after exercise, in trained men [109]. Likewise, an active session of isotonic knee extension resistance, including both concentric and eccentric phases (10 sets of 8 repetitions at 80% of 1RM) increased the expression of the BMAL1 gene by approximately 1.2 times, assessed 6 h after exercise in untrained healthy men [110], as well as positively regulating the clock genes Cry1 and Per2 by exercise, compared with control without exercise.

Four weeks of low-intensity resistance exercises resulted in a significant change in the expression of clock genes in the skeletal muscle of mice [111], supporting the fact that exercise can be an external stimulus for skeletal muscle rhythm. Skeletal muscle BMAL1 and Per2 gene expression significantly increased after a 12-week exercise intervention in elderly with obesity and prediabetes [112] Furthermore, skeletal muscle BMAL1 gene expression may improve insulin sensitivity. Interestingly, Clock- and BMAL1 gene mutant mice exhibit approximately 30% reductions in maximum muscle strength and 40% in mitochondrial volume [12]. Although with the absence of the CLOCK gene, the animals’ ability to adapt to 8 weeks of endurance exercise was not impaired [113].

Thus, the practice of physical exercise seems to modulate the interrupted skeletal muscle clock, contributing to improvements in the metabolic health of the entire body. These data have broad implications in the context of clinical practice, suggesting the importance of exercise, and, more specifically, the interaction of exercise and muscle, as a therapeutic strategy to help readjust body molecular clocks.

## 5. Impact of Exercise on Vascular Circadian Rhythm

The cardiovascular system is influenced to a great extent by chronobiologic rhythms that are determined by the CNS and peripheral clocks, determining short- (minutes and hours) and long-term (months and years) functional fluctuations. The peripheral clocks are within each cardiovascular cell and are crucial in the modulation of aspects such as endothelial function, vasodilation and resistance, blood pressure, hormone dynamics, body temperature, heart rate, etc. [114].

The effects of physical exercise on the cardiovascular system have been widely described, which include improvements in endothelial function, relaxation of the arterial wall and vasodilation, lower blood pressure and impedance, lower heart rate, improved heart-to-vascular coupling, and overall greater cardiovascular efficiency [115]. Even though acute exercise induces an increase in systolic blood pressure and heart rate, the intensification in shear stress that encompasses physical activity stimulates the release of NO by the endothelial cells, thus promoting vasodilation and improved blood supply to the working muscles [116,117,118]. The post-exercise phase depicts lower blood pressure and heart rate, in line with a positive modulation of the autonomic nervous system, with a change in the sympathetic/parasympathetic balance toward a higher influence of the parasympathetic axis [118]. The positive modulation of the cardiovascular system provided by physical exercise has also important long-term effects, mostly due to its beneficial impact on the endothelium and overall arterial structure, able to shift the trajectories of arterial aging toward a more beneficial one and therefore preventing the occurrence of early vascular aging [119,120]. Physical exercise thus provides a valuable tool to prevent biological aging and contribute to better cardiovascular health and lesser incidence of major cardiovascular events. Furthermore, physical exercise has been shown to modulate the circadian clocks to a similar extent as that produced by photic light cues [121], thus adding to its regulatory effect on the cardiovascular system. Physical exercise, particularly aerobic training, also produces important neuroendocrine changes, including, but not limited to, decreased cortisol and increased melatonin production during the night, contributing to a more effective sleep [122,123,124].

The adjustments of circadian rhythmicity produced by exercise have been shown to occur, even for low-intensity endurance exercise [111], and seem to be independent of the time of day the individual performs the exercise [125], although optimal diurnal exercise periods can be adjusted according to the individual chronotype [126]. According to previous research, individuals can be categorized into three distinct chronotype groups: early circadian chronotype, intermediate circadian chronotype, and late circadian chronotype [127]. Individuals in the early circadian chronotype group appear to have a greater disposition to early (morning) physical exercise, while those in the late circadian chronotype prefer to exercise during the late evening. The matching of exercise periodicity with individual chronotype could thus be an enhancement factor for skeletal muscle performance and circadian clock adjustments, producing better cardiovascular protection and improved sleep quality [128].

## 6. Conclusions

Mainly due to the pro-inflammatory cytokines impact of aging, there is a deregulation of the circadian rhythm, and its relationship with skeletal muscle changes; vascular health may be a possible risk factor for the development of sarcopenia. Lifestyle interventions such as regular physical exercise are essential to promoting an anti-inflammatory status, reducing muscle loss and strength, and improving endothelial function usually affected by aging. In addition, the adoption of physical exercise can act as a potential resynchronizer of peripheral muscle and vascular clock misalignment, which could favor vascular health and indirectly also affect positively skeletal muscle function. However, the exercise modality, as well as the ideal intensity and frequency to promote better effects on senescence, should be better investigated in future experimental studies.

## References

[1] López-Otín C., Blasco M.A., Partridge L., Serrano M., Kroemer G. (2013). The hallmarks of aging. Cell.

[2] Hood S., Amir S. (2017). The aging clock: Circadian rhythms and later life. J. Clin. Investig..

[3] Vitale J.A., Bonato M., La Torre A., Banfi G. (2019). The Role of the Molecular Clock in Promoting Skeletal Muscle Growth and Protecting against Sarcopenia. Int. J. Mol. Sci..

[4] Tahara Y., Shibata S. (2017). Entrainment of the mouse circadian clock: Effects of stress, exercise, and nutrition. Free. Radic. Biol. Med..

[5] Robinson I., Reddy A. (2014). Molecular mechanisms of the circadian clockwork in mammals. FEBS Lett..

[6] Kessel L., Lundeman J.H., Herbst K., Andersen T.V., Larsen M. (2010). Age-related changes in the transmission properties of the human lens and their relevance to circadian entrainment. J. Cataract. Refract. Surg..

[7] Arendt J. (2006). Melatonin and Human Rhythms. Chronobiol. Int..

[8] Hodge B.A., Wen Y., Riley L.A., Zhang X., England J.H., Harfmann B.D., Schroder E.A., Esser K. (2015). A The endogenous molecular clock orchestrates the temporal separation of substrate metabolism in skeletal muscle. Skelet. Muscle.

[9] Chatterjee S., Ma K. (2016). Circadian clock regulation of skeletal muscle growth and repair. F1000Research.

[10] Dyar K.A., Hubert M.J., Mir A.A., Ciciliot S., Lutter D., Greulich F., Quagliarini F., Kleinert M., Fischer K., Eichmann T.O. (2018). Transcriptional programming of lipid and amino acid metabolism by the skeletal muscle circadian clock. PLoS Biol..

[11] Fatima N., Rana S. (2020). Metabolic implications of circadian disruption. Pflug. Arch. Eur. J. Physiol..

[12] Andrews J.L., Zhang X., McCarthy J.J., McDearmon E.L., Hornberger T.A., Russell B., Campbell K.S., Arbogast S., Reid M.B., Walker J.R. (2010). CLOCK and BMAL1 regulate MyoD and are necessary for maintenance of skeletal muscle phenotype and function. Proc. Natl. Acad. Sci. USA.

[13] Li R., Xia J., Zhang X.I., Gathirua-Mwangi W.G., Guo J., Li Y., McKenzie S., Song Y. (2018). Associations of Muscle Mass and Strength with All-Cause Mortality among US Older Adults. Med. Sci. Sports Exerc..

[14] Nelke C., Dziewas R., Minnerup J., Meuth S.G., Ruck T. (2019). Skeletal muscle as potential central link between sarcopenia and immune senescence. EBioMedicine.

[15] Wang Y., Wehling-Henricks M., Welc S.S., Fisher A.L., Zuo Q., Tidball J.G. (2019). Aging of the immune system causes reductions in muscle stem cell populations, promotes their shift to a fibrogenic phenotype, and modulates sarcopenia. FASEB J. Off. Publ. Fed. Am. Soc. Exp. Biol..

[16] Watanabe S., Kawamoto S., Ohtani N., Hara E. (2017). Impact of senescence-associated secretory phenotype and its potential as a therapeutic target for senescence-associated diseases. Cancer Sci..

[17] Donato A.J., Machin D.R., Lesniewski L.A. (2018). Mechanisms of Dysfunction in the Aging Vasculature and Role in Age-Related Disease. Circ. Res..

[18] Addison O., Prior S.J., Kundi R., Serra M.C., Katzel L.I., Gardner A.W., Ryan A.S. (2018). Sarcopenia in Peripheral Arterial Disease: Prevalence and Effect on Functional Status. Arch. Phys. Med. Rehabil..

[19] McAlpine C.S., Swirski F.K. (2016). Circadian Influence on Metabolism and Inflammation in Atherosclerosis. Circ. Res..

[20] Cruz-Jentoft A.J., Bahat G., Bauer J., Boirie Y., Bruyère O., Cederholm T., Cooper C., Landi F., Rolland Y., Sayer A.A. (2019). Sarcopenia: Revised European consensus on definition and diagnosis. Age Ageing.

[21] Cruz-Jentoft A.J., Baeyens J.P., Bauer J.M., Boirie Y., Cederholm T., Landi F., Martin F.C., Michel J.-P., Rolland Y., Schneider S.M. (2010). Sarcopenia: European consensus on definition and diagnosis: Report of the European Working Group on Sarcopenia in Older People. Age Ageing.

[22] Mijnarends D.M., Koster A., Schols J.M.G.A., Meijers J.M.M., Halfens R.J.G., Gudnason V., Eiriksdottir G., Siggeirsdottir K., Sigurdsson S., Jónsson P.V. (2016). Physical activity and incidence of sarcopenia: The population-based AGES-Reykjavik Study. Age Ageing.

[23] Volpi E., Nazemi R., Fujita S. (2004). Muscle tissue changes with aging. Curr. Opin. Clin. Nutr. Metab. Care.

[24] Leonardi G.C., Accardi G., Monastero R., Nicoletti F., Libra M. (2018). Ageing: From inflammation to cancer. Immun. Ageing.

[25] Ruparelia N., Chai J.T., Fisher E.A., Choudhury R.P. (2016). Inflammatory processes in cardiovascular disease: A route to targeted therapies. Nat. Rev. Cardiol..

[26] Salimi S., Shardell M.D., Seliger S.L., Bandinelli S., Guralnik J.M., Ferrucci L. (2018). Inflammation and Trajectory of Renal Function in Community-Dwelling Older Adults. J. Am. Geriatr. Soc..

[27] Bano G., Trevisan C., Carraro S., Solmi M., Luchini C., Stubbs B., Manzato E., Sergi G., Veronese N. (2017). Inflammation and sarcopenia: A systematic review and meta-analysis. Maturitas.

[28] Cipolletta D., Cohen P., Spiegelman B.M., Benoist C., Mathis D. (2015). Appearance and disappearance of the mRNA signature characteristic of Treg cells in visceral adipose tissue: Age, diet, and PPARγ effects. Proc. Natl. Acad. Sci. USA.

[29] Macdougall C.E., Wood E.G., Loschko J., Scagliotti V., Cassidy F.C., Robinson M.E., Feldhahn N., Castellano L., Voisin M.-B., Marelli-Berg F. (2018). Visceral Adipose Tissue Immune Homeostasis Is Regulated by the Crosstalk between Adipocytes and Dendritic Cell Subsets. Cell Metab..

[30] McHugh D., Gil J. (2017). Senescence and aging: Causes, consequences, and therapeutic avenues. J. Cell Biol..

[31] Tchkonia T., Zhu Y., van Deursen J., Campisi J., Kirkland J.L. (2013). Cellular senescence and the senescent secretory phenotype: Therapeutic opportunities. J. Clin. Investig..

[32] Visser M., Pahor M., Taaffe D.R., Goodpaster B.H., Simonsick E.M., Newman A.B., Nevitt M., Harris T.B. (2002). Relationship of Interleukin-6 and Tumor Necrosis Factor-α With Muscle Mass and Muscle Strength in Elderly Men and Women: The Health ABC Study. J. Gerontol. Ser. A.

[33] Meng S.-J., Yu L.-J. (2010). Oxidative Stress, Molecular Inflammation and Sarcopenia. Int. J. Mol. Sci..

[34] Cielen N., Maes K., Gayan-Ramirez G. (2014). Musculoskeletal Disorders in Chronic Obstructive Pulmonary Disease. BioMed Res. Int..

[35] Bodine S.C., Edward F. (2020). Adolph Distinguished Lecture. Skeletal muscle atrophy: Multiple pathways leading to a common outcome. J. Appl. Physiol..

[36] Li Y.P., Chen Y., John J., Moylan J., Jin B., Mann D.L., Reid M.B. (2005). TNF-alpha acts via p38 MAPK to stimulate expression of the ubiquitin ligase atrogin1/MAFbx in skeletal muscle. FASEB J..

[37] Hershko A. (2005). The ubiquitin system for protein degradation and some of its roles in the control of the cell division cycle. Cell Death Differ..

[38] Madaro L., Passafaro M., Sala D., Etxaniz U., Lugarini F., Proietti D., Alfonsi M.V., Nicoletti C., Gatto S., De Bardi M. (2018). Denervation-activated STAT3-IL-6 signalling in fibro-adipogenic progenitors promotes myofibres atrophy and fibrosis. Nature.

[39] Pedersen B.K., Febbraio M.A. (2008). Muscle as an Endocrine Organ: Focus on Muscle-Derived Interleukin-6. Physiol. Rev..

[40] Saxena A., Khosraviani S., Noel S., Mohan D., Donner T., Hamad A.R.A. (2015). Interleukin-10 paradox: A potent immunoregulatory cytokine that has been difficult to harness for immunotherapy. Cytokine.

[41] Chazaud B., Brigitte M., Yacoub-Youssef H., Arnold L., Gherardi R., Sonnet C., Lafuste P., Chretien F. (2009). Dual and Beneficial Roles of Macrophages During Skeletal Muscle Regeneration. Exerc. Sport Sci. Rev..

[42] Tidball J.G., Villalta S.A. (2010). Regulatory interactions between muscle and the immune system during muscle regeneration. Am. J. Physiol. Regul. Integr. Comp. Physiol..

[43] Saclier M., Cuvellier S., Magnan M., Mounier R., Chazaud B. (2013). Monocyte/macrophage interactions with myogenic precursor cells during skeletal muscle regeneration. FEBS J..

[44] Mantovani A., Sica A., Sozzani S., Allavena P., Vecchi A., Locati M. (2004). The chemokine system in diverse forms of macrophage activation and polarization. Trends Immunol..

[45] Al-Dabbagh S., McPhee J.S., Murgatroyd C., Butler-Browne G., Stewart C.E., Al-Shanti N. (2015). The lymphocyte secretome from young adults enhances skeletal muscle proliferation and migration, but effects are attenuated in the secretome of older adults. Physiol. Rep..

[46] Zhang J., Xiao Z., Qu C., Cui W., Wang X., Du J. (2014). CD8 T cells are involved in skeletal muscle regeneration through facilitating MCP-1 secretion and Gr1(high) macrophage infiltration. J. Immunol..

[47] Schiaffino S., Pereira M.G., Ciciliot S., Rovere-Querini P. (2016). Regulatory T cells and skeletal muscle regeneration. FEBS J..

[48] Costamagna D., Costelli P., Sampaolesi M., Penna F. (2015). Role of Inflammation in Muscle Homeostasis and Myogenesis. Mediat. Inflamm..

[49] Reidy P.T., McKenzie A.I., Mahmassani Z., Petrocelli J., Nelson D.B., Lindsay C.C., Gardner J.E., Morrow V.R., Keefe A.C., Huffaker T.B. (2019). Aging impairs mouse skeletal muscle macrophage polarization and muscle-specific abundance during recovery from disuse. Am. J. Physiol. Metab..

[50] Duggal N.A., Pollock R.D., Lazarus N.R., Harridge S., Lord J.M. (2018). Major features of immunesenescence, including reduced thymic output, are ameliorated by high levels of physical activity in adulthood. Aging Cell.

[51] Cavadini G., Petrzilka S., Kohler P., Jud C., Tobler I., Birchler T., Fontana A. (2007). TNF- suppresses the expression of clock genes by interfering with E-box-mediated transcription. Proc. Natl. Acad. Sci. USA.

[52] Yoshida K., Nakai A., Kaneshiro K., Hashimoto N., Suzuki K., Uchida K., Hashimoto T., Kawasaki Y., Tateishi K., Nakagawa N. (2018). TNF-α induces expression of the circadian clock gene Bmal1 via dual calcium-dependent pathways in rheumatoid synovial cells. Biochem. Biophys. Res. Commun..

[53] Vieira E., Mirizio G.G. (2020). Clock Genes, Inflammation and the Immune System-Implications for Diabetes, Obesity and Neurodegenerative Diseases. Int. J. Mol. Sci..

[54] Spengler M.L., Kuropatwinski K.K., Comas M., Gasparian A.V., Fedtsova N., Gleiberman A.S., Gitlin I.I., Artemicheva N.M., Deluca K.A., Gudkov A.V. (2012). Core circadian protein CLOCK is a positive regulator of NF-κB-mediated transcription. Proc. Natl. Acad. Sci. USA.

[55] Narasimamurthy R., Hatori M., Nayak S.K., Liu F., Panda S., Verma I.M. (2012). Circadian clock protein cryptochrome regulates the expression of proinflammatory cytokines. Proc. Natl. Acad. Sci. USA.

[56] Schroder E.A., Harfmann B.D., Zhang X., Srikuea R., England J.H., Hodge B.A., Wen Y., Riley L.A., Yu Q., Christie A. (2015). Intrinsic muscle clock is necessary for musculoskeletal health. J. Physiol..

[57] Kondratov R.V., Kondratova A.A., Gorbacheva V.Y., Vykhovanets O.V., Antoch M.P. (2006). Early aging and age-related pathologies in mice deficient in BMAL1, the core componentof the circadian clock. Genes Dev..

[58] Liu J., Zhou B., Yan M., Huang R., Wang Y., He Z., Yang Y., Dai C., Wang Y., Zhang F. (2016). CLOCK and BMAL1 Regulate Muscle Insulin Sensitivity via SIRT1 in Male Mice. Endocrinology.

[59] Choi Y.I., Park D.K., Chung J.-W., Kim K.O., Kwon K.A., Kim Y.J. (2019). Circadian rhythm disruption is associated with an increased risk of sarcopenia: A nationwide population-based study in Korea. Sci. Rep..

[60] Ben-Shlomo Y., Spears M., Boustred C., May M., Anderson S.G., Benjamin E.J., Boutouyrie P., Cameron J., Chen C.H., Cruickshank J.K. (2014). Aortic pulse wave velocity improves cardiovascular event prediction: An individual participant meta-analysis of prospective observational data from 17,635 subjects. J. Am. Coll. Cardiol..

[61] Abbatecola A.M., Chiodini P., Gallo C., Lakatta E., Sutton-Tyrrell K., Tylavsky F.A., Goodpaster B., de Rekeneire N., Schwartz A.V., Paolisso G. (2011). Pulse wave velocity is associated with muscle mass decline: Health ABC study. AGE.

[62] Kohara K., Okada Y., Ochi M., Ohara M., Nagai T., Tabara Y., Igase M. (2017). Muscle mass decline, arterial stiffness, white matter hyperintensity, and cognitive impairment: Japan Shimanami Health Promoting Program study. J. Cachex-Sarcopenia Muscle.

[63] Dvoretskiy S., Lieblein-Boff J.C., Jonnalagadda S., Atherton P.J., Phillips B.E., Pereira S.L. (2020). Exploring the Association between Vascular Dysfunction and Skeletal Muscle Mass, Strength and Function in Healthy Adults: A Systematic Review. Nutrients.

[64] Jeon Y.K., Shin M.J., Saini S.K., Custodero C., Aggarwal M., Anton S.D., Leeuwenburgh C., Mankowski R.T. (2021). Vascular dysfunction as a potential culprit of sarcopenia. Exp. Gerontol..

[65] Hamczyk M.R., Nevado R.M., Barettino A., Fuster V., Andres V. (2020). Biological Versus Chronological Aging: JACC Focus Seminar. J. Am. Coll. Cardiol..

[66] Ungvari Z., Tarantini S., Sorond F., Merkely B., Csiszar A. (2020). Mechanisms of Vascular Aging, A Geroscience Perspective: JACC Focus Seminar. J. Am. Coll. Cardiol..

[67] Incalza M.A., D’Oria R., Natalicchio A., Perrini S., Laviola L., Giorgino F. (2018). Oxidative stress and reactive oxygen species in endothelial dysfunction associated with cardiovascular and metabolic diseases. Vasc. Pharmacol..

[68] Zhang W.J., Li P.X., Guo X.H., Huang Q.B. (2017). Role of moesin, Src, and ROS in advanced glycation end product-induced vascular endothelial dysfunction. Microcirculation.

[69] Zhao M.M., Xu M.J., Cai Y., Zhao G., Guan Y., Kong W., Tang C., Wang X. (2011). Mitochondrial reactive oxygen species promote p65 nuclear translocation mediating high-phosphate-induced vascular calcification in vitro and in vivo. Kidney Int..

[70] Takemura A., Iijima K., Ota H., Son B.K., Ito Y., Ogawa S., Eto M., Akishita M., Ouchi Y. (2011). Sirtuin 1 Retards Hyperphosphatemia-Induced Calcification of Vascular Smooth Muscle Cells. Arter. Thromb. Vasc. Biol..

[71] Xiao X., Yang C., Qu S.-L., Shao Y.-D., Zhou C.-Y., Chao R., Huang L., Zhang C. (2019). S100 proteins in atherosclerosis. Clin. Chim. Acta.

[72] Al-Aly Z. (2011). Phosphate, oxidative stress, and nuclear factor-κB activation in vascular calcification. Kidney Int..

[73] Nadra I., Mason J.C., Philippidis P., Florey O., Smythe C.D., McCarthy G.M., Landis R.C., Haskard D.O. (2005). Proinflammatory activation of macrophages by basic calcium phosphate crystals via protein kinase C and MAP kinase pathways: A vicious cycle of inflammation and arterial calcification?. Circ. Res..

[74] Tintut Y., Patel J., Parhami F., Demer L.L. (2000). Tumor Necrosis Factor-α Promotes In Vitro Calcification of Vascular Cells via the cAMP Pathway. Circulation.

[75] Duncan E.R., Crossey P.A., Walker S., Anilkumar N., Poston L., Douglas G., Ezzat V.A., Wheatcroft S.B., Shah A.M., Kearney M.T. (2008). Effect of endothelium-specific insulin resistance on endothelial function in vivo. Diabetes.

[76] Wilkes E.A., Selby A.L., Atherton P.J., Patel R., Rankin D., Smith K., Rennie M.J. (2009). Blunting of insulin inhibition of proteolysis in legs of older subjects may contribute to age-related sarcopenia. Am. J. Clin. Nutr..

[77] Chen L.K., Liu L.K., Woo J., Assantachai P., Auyeung T.W., Bahyah K.S., Chou M.Y., Chen L.Y., Hsu P.S., Krairit O. (2014). Sarcopenia in Asia: Consensus Report of the Asian Working Group for Sarcopenia. J. Am. Med. Dir. Assoc..

[78] Pereira T., Cipriano I., Costa T., Saraiva M., Martins A., Consortium A.G.L. (2019). Exercise, ageing and cognitive function—Effects of a personalized physical exercise program in the cognitive function of older adults. Physiol. Behav..

[79] Tang A., Eng J.J., Brasher P.M., Madden K.M., Mohammadi A., Krassioukov A.V., Tsang T.S. (2014). Physical Activity Correlates with Arterial Stiffness in Community-dwelling Individuals with Stroke. J. Stroke Cerebrovasc. Dis..

[80] Zanetti H.R., Mendes E.L., Gonçalves A., Lopes L.T., Roever L., Silva-Vergara M.L., Neves F.F., Resende E.S. (2020). Effects of exercise training and statin on hemodynamic, biochemical, inflammatory and immune profile of people living with HIV: A randomized, double-blind, placebo-controlled trial. J. Sports Med. Phys. Fit..

[81] Teixeira M., Gouveia M., Duarte A., Ferreira M., Simoes M.I., Conceicao M., Silva G., Magalhaes S., Ferreira R., Nunes A. (2019). Regular Exercise Participation Contributes to Better Proteostasis, Inflammatory Profile, and Vasoactive Profile in Patients With Hypertension. Am. J. Hypertens..

[82] Lavin K.M., Perkins R.K., Jemiolo B., Raue U., Trappe S.W., Trappe T.A. (2020). Effects of aging and lifelong aerobic exercise on basal and exercise-induced inflammation. J. Appl. Physiol..

[83] Despeghel M., Reichel T., Zander J., Krüger K., Weyh C. (2021). Effects of a 6 Week Low-Dose Combined Resistance and Endurance Training on T Cells and Systemic Inflammation in the Elderly. Cells.

[84] Mela V., Mota B.C., Milner M., McGinley A., Mills K.H.G., Kelly A.M., Lynch M.A. (2020). Exercise-induced re-programming of age-related metabolic changes in microglia is accompanied by a reduction in senescent cells. Brain Behav. Immun..

[85] Mathot E., Liberman K., Cao Dinh H., Njemini R., Bautmans I. (2021). Systematic review on the effects of physical exercise on cellular immunosenescence-related markers—An update. Exp. Gerontol..

[86] Zwetsloot M.J., Bauerle T.L. (2021). Repetitive seasonal drought causes substantial species-specific shifts in fine-root longevity and spatio-temporal production patterns in mature temperate forest trees. New Phytol..

[87] Duggal N.A., Niemiro G., Harridge S.D.R., Simpson R.J., Lord J.M. (2019). Can physical activity ameliorate immunosenescence and thereby reduce age-related multi-morbidity?. Nat. Rev. Immunol..

[88] Farinha J.B., Steckling F.M., Stefanello S.T., Cardoso M.S., Nunes L.S., Barcelos R.P., Duarte T., Kretzmann N.A., Mota C.B., Bresciani G. (2015). Response of oxidative stress and inflammatory biomarkers to a 12-week aerobic exercise training in women with metabolic syndrome. Sports Med. Open.

[89] Chen L., Bai J., Li Y. (2020). The Change of Interleukin-6 Level-Related Genes and Pathways Induced by Exercise in Sedentary Individuals. J. Interf. Cytokine Res..

[90] Svajger U., Tesic N., Rozman P. (2021). Programmed death ligand 1 (PD-L1) plays a vital part in DC tolerogenicity induced by IFN-gamma. Int. Immunopharmacol..

[91] Rozman P., Svajger U. (2018). The tolerogenic role of IFN-gamma. Cytokine Growth Factor Rev..

[92] Shaw D.M., Merien F., Braakhuis A., Keaney L., Dulson D.K. (2020). Acute hyperketonaemia alters T-cell-related cytokine gene expression within stimulated peripheral blood mononuclear cells following prolonged exercise. Eur. J. Appl. Physiol..

[93] Hasanli S., Hojjati S., Koushkie Jahromi M. (2020). The Effect of Exercise and Psychological Stress on Anti- and Proinflammatory Cytokines. Neuroimmunomodulation.

[94] Vijayaraghava A. (2017). Behavior of plasma interferon-gamma with graded exercise in individuals with varied body mass index and age: Risk stratification of predisposition to inflammation. Natl. J. Physiol. Pharm. Pharmacol..

[95] Papini C.B., Nakamura P.M., Zorzetto L.P., Thompson J.L., Phillips A.C., Kokubun E. (2014). The Effect of a Community-Based, Primary Health Care Exercise Program on Inflammatory Biomarkers and Hormone Levels. Mediat. Inflamm..

[96] Liberman K., Forti L.N., Beyer I., Bautmans I. (2017). The effects of exercise on muscle strength, body composition, physical functioning and the inflammatory profile of older adults: A systematic review. Curr. Opin. Clin. Nutr. Metab. Care.

[97] Afsin A., Bozyilan E., Asoglu R., Hosoglu Y., Dundar A.A. (2021). Effects of regular exercise on inflammatory biomarkers and lipid parameters in soccer players. J. Immunoass. Immunochem..

[98] Venkatesh A., Edirappuli S.D., Zaman H.P., Zaman R. (2020). The Effect of Exercise on Mental Health: A Focus on Inflammatory Mechanisms. Psychiatr. Danub..

[99] González-Gil E.M., Santaliestra-Pasías A.M., Buck C., Gracia-Marco L., Lauria F., Pala V., Molnar D., Veidebaum T., Iacoviello L., Tornaritis M. (2021). Improving cardiorespiratory fitness protects against inflammation in children: The IDEFICS study. Pediatr. Res..

[100] Bautmans I., Salimans L., Njemini R., Beyer I., Lieten S., Liberman K. (2021). The effects of exercise interventions on the inflammatory profile of older adults: A systematic review of the recent literature. Exp. Gerontol..

[101] Franceschi C., Garagnani P., Parini P., Giuliani C., Santoro A. (2018). Inflammaging: A new immune–metabolic viewpoint for age-related diseases. Nat. Rev. Endocrinol..

[102] Patterson R.E., Sears D.D. (2017). Metabolic effects of intermittent fasting. Annu. Rev. Nutr..

[103] Wolff A.C., Esser A.K. (2019). Exercise timing and circadian rhythms. Curr. Opin. Physiol..

[104] Dickinson J.M., D’Lugos A.C., Naymik M.A., Siniard A.L., Wolfe A.J., Curtis D.R., Huentelman M.J., Carroll C.C. (2018). Transcriptome response of human skeletal muscle to divergent exercise stimuli. J. Appl. Physiol..

[105] Lassiter D.G., Sjogren R.J.O., Gabriel B.M., Krook A., Zierath J.R. (2018). AMPK activation negatively regulates GDAP1, which influences metabolic processes and circadian gene expression in skeletal muscle. Mol. Metab..

[106] Peek C.B., Levine D.C., Cedernaes J., Taguchi A., Kobayashi Y., Tsai S.J., Bonar N.A., McNulty M.R., Ramsey K.M., Bass J. (2016). Circadian Clock Interaction with HIF1alpha Mediates Oxygenic Metabolism and Anaerobic Glycolysis in Skeletal Muscle. Cell Metab..

[107] de Souza Teixeira A.A., Minuzzi L.G., Lira F.S., Goncalves A., Martinho A., Rosa Neto J.C., Teixeira A.M. (2021). Improvement in the anti-inflammatory profile with lifelong physical exercise is related to clock genes expression in effector-memory CD4+ T cells in master athletes. Exerc. Immunol. Rev..

[108] Tylutka A., Morawin B., Gramacki A., Zembron-Lacny A. (2021). Lifestyle exercise attenuates immunosenescence; flow cytometry analysis. BMC Geriatr..

[109] Popov D.V., Makhnovskii P.A., Kurochkina N.S., Lysenko E.A., Vepkhvadze T.F., Vinogradova O.L. (2018). Intensity-dependent gene expression after aerobic exercise in endurance-trained skeletal muscle. Biol. Sport.

[110] Zambon A.C., McDearmon E.L., Salomonis N., Vranizan K.M., Johansen K.L., Adey D., Takahashi J.S., Schambelan M., Conklin B.R. (2003). Time- and exercise-dependent gene regulation in human skeletal muscle. Genome Biol..

[111] Wolff G., Esser K.A. (2012). Scheduled Exercise Phase Shifts the Circadian Clock in Skeletal Muscle. Med. Sci. Sports Exerc..

[112] Erickson M.L., Zhang H., Mey J.T., Kirwan J.P. (2020). Exercise Training Impacts Skeletal Muscle Clock Machinery in Prediabetes. Med. Sci. Sports Exerc..

[113] Pastore S., Hood D.A. (2013). Endurance training ameliorates the metabolic and performance characteristics of circadian Clock mutant mice. J. Appl. Physiol..

[114] Crnko S., Du Pre B.C., Sluijter J.P.G., Van Laake L.W. (2019). Circadian rhythms and the molecular clock in cardiovascular biology and disease. Nat. Rev. Cardiol..

[115] Fiuza-Luces C., Santos-Lozano A., Joyner M., Carrera-Bastos P., Picazo O., Zugaza J.L., Izquierdo M., Ruilope L.M., Lucia A. (2018). Exercise benefits in cardiovascular disease: Beyond attenuation of traditional risk factors. Nat. Rev. Cardiol..

[116] Green D.J., Smith K.J. (2017). Effects of Exercise on Vascular Function, Structure, and Health in Humans. Cold Spring Harb. Perspect. Med..

[117] Green D.J., Hopman M.T.E., Padilla J., Laughlin M.H., Thijssen D.H.J. (2017). Vascular Adaptation to Exercise in Humans: Role of Hemodynamic Stimuli. Physiol. Rev..

[118] Cornelissen V.A., Verheyden B., Aubert E.A., Fagard R.H. (2010). Effects of aerobic training intensity on resting, exercise and post-exercise blood pressure, heart rate and heart-rate variability. J. Hum. Hypertens..

[119] Ross M.D., Malone E., Florida-James G. (2015). Vascular Ageing and Exercise: Focus on Cellular Reparative Processes. Oxidative Med. Cell. Longev..

[120] Nilsson P.M. (2008). Early vascular aging (EVA): Consequences and prevention. Vasc. Health Risk Manag..

[121] Yamanaka Y., Hashimoto S., Masubuchi S., Natsubori A., Nishide S.-Y., Honma S., Honma K.-I. (2014). Differential regulation of circadian melatonin rhythm and sleep-wake cycle by bright lights and nonphotic time cues in humans. Am. J. Physiol. Regul. Integr. Comp. Physiol..

[122] Leonardo-Mendonça R.C., Martinez-Nicolas A., Galván C.D.T., Ocaña-Wilhelmi J., Rusanova I., Guerra-Hernández E., Escames G., Acuña-Castroviejo D. (2015). The benefits of four weeks of melatonin treatment on circadian patterns in resistance-trained athletes. Chronobiol. Int..

[123] Hackney A.C., Davis H.C., Lane A.R. (2015). Exercise augments the nocturnal prolactin rise in exercise-trained men. Ther. Adv. Endocrinol. Metab..

[124] Heaney J.L., Carroll D., Whittaker A. (2014). Physical Activity, Life Events Stress, Cortisol, and DHEA: Preliminary Findings That Physical Activity May Buffer Against the Negative Effects of Stress. J. Aging Phys. Act..

[125] Hower I.M., Harper S.A., Buford T.W. (2018). Circadian Rhythms, Exercise, and Cardiovascular Health. J. Circadian Rhythm..

[126] Rossi A., Formenti D., Vitale J.A., Calogiuri G., Weydahl A. (2015). The Effect of Chronotype on Psychophysiological Responses during Aerobic Self-Paced Exercises. Percept. Mot. Ski..

[127] Facer-Childs E.R., Brandstaetter R. (2015). The Impact of Circadian Phenotype and Time since Awakening on Diurnal Performance in Athletes. Curr. Biol..

[128] Fairbrother K., Cartner B., Alley J.R., Curry C.D., Dickinson D.L., Morris D.M., Collier S.R. (2014). Effects of exercise timing on sleep architecture and nocturnal blood pressure in prehypertensives. Vasc. Health Risk Manag..

